# Inulin supplementation exhibits increased muscle mass via gut-muscle axis in children with obesity: double evidence from clinical and in vitro studies

**DOI:** 10.1038/s41598-024-61781-1

**Published:** 2024-05-16

**Authors:** Chonnikant Visuthranukul, Asada Leelahavanichkul, Surapun Tepaamorndech, Supakarn Chamni, Eakkarin Mekangkul, Sirinuch Chomtho

**Affiliations:** 1 Pediatric Nutrition Research Unit, Division of Nutrition, Department of Pediatrics, Faculty of Medicine, Chulalongkorn University, 1873 Rama IV Road, Pathumwan, Bangkok, 10330 Thailand; 2 Department of Microbiology, Faculty of Medicine, Chulalongkorn University, Bangkok, 10330 Thailand; 3 Center of Excellence in Inflammation and Immunology Research Unit (CETRII), Department of Microbiology, Chulalongkorn University, Bangkok, 10330 Thailand; 4 Natural Products and Nanoparticles Research Unit (NP2), Department of Pharmacognosy and Pharmaceutical Botany, Faculty of Pharmaceutical Sciences, Chulalongkorn University, Bangkok, 10330 Thailand

**Keywords:** Gut-muscle axis, Muscle biomarkers, Fat-free mass gain, Inflammatory cytokines, Anti-inflammatory markers, Children with obesity, Microbiology, Medical research, Diseases, Nutrition disorders

## Abstract

Gut microbiota manipulation may reverse metabolic abnormalities in obesity. Our previous studies demonstrated that inulin supplementation significantly promoted *Bifidobacterium* and fat-free mass in obese children. We aimed to study gut-muscle axis from inulin supplementation in these children. In clinical phase, the plasma samples from 46 participants aged 7–15 years, were analyzed for muscle biomarkers before and after 6-month inulin supplementation. In parallel, the plausible mechanism of muscle production via gut-muscle axis was examined using macrophage cell line. *Bifidobacterium* was cultured in semi-refined medium with inulin used in the clinical phase. Cell-free supernatant was collected and used in lipopolysaccharide (LPS)-induced macrophage cell line to determine inflammatory and anti-inflammatory gene expression. In clinical phase, IL-15 and creatinine/cystatin C ratio significantly increased from baseline to the 6th month. In vitro study showed that metabolites derived from *Bifidobacterium* capable of utilizing inulin contained the abundance of SCFAs. In the presence of LPS, treatment from *Bifidobacterium* + inulin downregulated *TNF-α*, *IL-6*, *IL-1β*, and *iNOS*, but upregulated *FIZZ-1* and *TGF-β* expression. Inulin supplementation promoted the muscle biomarkers in agreement with fat-free mass gain, elucidating by *Bifidobacterium* metabolites derived from inulin digestion showed in vitro anti-inflammatory activity and decreased systemic pro-inflammation, thus promoting muscle production via gut-muscle axis response.

Clinical Trial Registry number: NCT03968003.

## Introduction

Obesity is widely recognized as one of the leading causes of non-communicable diseases such as diabetes mellitus, cardiovascular diseases, non-alcoholic fatty liver disease (NAFLD), cancer, and impairment of immunity^1^. The global prevalence of childhood overweight and obesity stood at 6.7% in 2010 and exhibited a trajectory toward reaching 9.1% by 2020^2^. Obese children's behaviors have an impact on their eating styles, such as an increased enjoyment of food, and they tend to prefer low-fiber diets and high sugary foods like sweetened drinks, such as fruit juices with varying glycemic load, contributing to obesity^3,4^. Therefore, providing suitable dietary management for children with obesity is crucial.

Presently, obesity is considered a chronic inflammatory condition with several supporting hypotheses. Lipopolysaccharide (LPS), a membrane component of gram-negative bacteria, is supposed to be one of the triggers in pathogenesis. LPS translocation across the intestinal barrier triggers inflammatory responses. LPS binds to plasma LPS-binding proteins, thereby triggering toll-like receptor 4 (TLR4) activation on the surface of macrophages within adipose tissue. This initiation stimulates the genes encoding nuclear factor-κB (NF-κB), which subsequently induce the production of inflammatory cytokines. Furthermore, LPS is involved in the inflammasome pathway, leading to the induction of interleukin-1β (IL-1β). Together, these processes contribute to the development of chronic, low-grade inflammation, a condition strongly linked to obesity and metabolic syndrome^5^. High-fat diets have also been observed to increase LPS absorption in the intestine, which is relevant as children with obesity often consume such high-fat diets, thereby increasing their risk of metabolic complications. Additionally, dysbiosis of the gut microbiota can disrupt intestinal permeability by reducing beneficial bacteria, like *Bifidobacterium*^1,6,7^. All these factors aggravate gram-negative bacteria which induce absorption of LPS to systemic circulation, resulting in the chronic inflammation in obesity.

To improve the intestinal microbial ecosystem, prebiotics, non-digestible polysaccharides have been studied and shown promising results in promoting a healthy balance of gut microbiota when supplemented in obese children^1,8^. Inulin, a fructan-type polysaccharide, is renowned as an evidence-based prebiotic. Several studies found inulin supplementation in individuals with obesity promoted the growth of *Bifidobacterium* spp. in the intestine^9,10^, decreases in body weight and body mass index (BMI)^11^, decreases in percent body and trunk fat^10^, an improvement in satiety and reductions in postprandial glucose and insulin levels^12^.

Recently, we conducted a randomized, double-blinded placebo-controlled study of inulin supplementation extracted from Thai Jerusalem artichoke using our patented technology in obese children (Patent no. 15858, Inventor: Chonnikant Visuthranukul and Supakarn Chamni, Chulalongkorn University and National Science and Technology Development Agency, Thailand). Of one hundred and fifty-five participants; interestingly, we found a significant increase in fat-free mass and *Bifidobacterium* only in the inulin group^13^. Improving the intestinal microbial ecosystem is believed to reduce pro-inflammation^10^ while enhance anti-inflammatory activity. It was already known that *Bifidobacterium* utilizes inulin to yield substrates essential for other short-chain fatty acid (SCFA)-producing bacteria, thereby contributing to the production of SCFAs^14^. Therefore, we hypothesized that metabolites-derived from *Bifidobacterium* would exhibit anti-inflammatory activity leading to myogenesis by increasing mitochondrial biogenesis, capillary density, glycogen storage, and protein synthesis in muscle mass^15^. Based on our previous finding in fat-free mass gain^13^, there has not been research done in the aspect of gut-muscle axis response in the pediatric obesity. To further underpin this knowledge, we aimed to study gut-muscle axis from inulin supplementation on muscle mass in this population.

## Materials and methods

### Subjects

This present study used the plasma and serum samples from the previously published randomized double-blinded placebo-controlled trial which conducted from August 2017 to July 2020 at the King Chulalongkorn Memorial Hospital (KCMH), Thailand^13^. The Institutional Review Board of the Faculty of Medicine, Chulalongkorn University approved the study protocol (IRB No. 240/60). All experiments were performed in accordance with relevant guidelines and regulations. Informed consent was obtained from all subjects and their legal guardians prior to study enrollment. This trial was registered at clinicaltrials.gov (NCT03968003). Thai children aged 7 to 15 years with obesity as a BMI more than 2 standard deviations (SDs) above median as per the WHO growth reference^16^ were recruited as mentioned in the previous study^13^.

### Study design

The detailed study design has been published elsewhere^13^. In brief, Thai children aged 7–15 years with obesity were randomly assigned to inulin extracted from Jerusalem artichoke by our patented technique (intervention), maltodextrin (placebo), and dietary fiber advice groups. All participants received monthly follow-up with the same standard advice of dietary intake and exercise for 6 months. The details and flow diagram of the study are reported elsewhere^13^.

### The in vivo biomarkers of muscle building

The stored plasma and serum samples from the randomised controlled trial (RCT) of inulin supplementation in obese children were analyzed for biomarkers of muscle building at the 1st and 6th visits. Plasma and serum samples were stored at − 80 °C until analysis. For the present study, 56 plasma/serum samples (n = 46 from the inulin group, n = 5 from each placebo and dietary fiber advice group) were analyzed for myokine and creatinine/cystatin C ratio to support the results of muscle building from the previous study^13^. Myokine, interleukin-15 (IL-15), was analyzed by enzyme-linked immunosorbent assay (ELISA). The quantification of IL-15, using the respective Human IL-15 ELISA kit (ab218266, Abcam, UK), was conducted in accordance with the prescribed manufacturer's protocols. Briefly, 50 µL of the sample was introduced into each well, followed by the addition of 50 µL of antibody, and incubation at room temperature for one hour. Subsequently, the sample mixture underwent three wash cycles using 350 µL of 1X wash buffer per cycle prior to the introduction of 100 µL of TMB development solution. The reaction proceeded at room temperature for 10 min and was subsequently terminated by the addition of 100 µL of stop solution. Measurement of the absorbance was carried out at a wavelength of 450 nm using a microplate reader. Furthermore, the concentration of creatinine in serum was measured using an enzymatic method on the Alinity C analyzers (Abbott Laboratories, USA), and that of cystatin C, N Latex Cystatin C, is an in vitro diagnostics kit containing reagents for the quantitative determination of cystatin C in human serum by means of particle-enhanced immunonephelometry using the BN Systems (SIEMENS, USA) with blood specimens stored at − 80 °C from the baseline and final visit. The samples were automatically diluted 1:100 with N Diluent and then were measured within four hours. If the results obtained were outside the measuring range, the assay was repeated using a higher or lower dilution of the sample.

### The in vitro experiments

In parallel, the plausible mechanism of muscle mass building in the obese participants via gut-muscle axis was examined for in vitro phase. To test an influence of the metabolite from *Bifidobacterium* with or without inulin against macrophages, the condition media from *Bifidobacterium* (*Bifidobacterium* condition media; Bifido) with or without inulin were tested in a macrophage cell line (RAW264.7).

In Bifido preparation, *Bifidobacterium longum* HFDGO02 was cultured in SM medium using either glucose or inulin as the carbon source. Briefly, SM medium, glucose, and inulin were separately autoclaved. The medium containing either 1% (W/V) glucose or inulin was prepared and used for bacterial culture at 37 °C under an anaerobic condition for 48 h^17^. The supernatants were then collected by centrifugation and filtered (0.22-µm membrane filter) (Minisart; Sartorius Stedim Biotech GmbH, Göttingen, Germany), and 500 µl of the preparation was concentrated by speed vacuum drying at 40 °C for 3 h (Savant Instruments, Farmingdale, NY). The cell-free concentrated pellets were resuspended in an equal volume of DMEM or further diluted by DMEM into 50 and 25% dilution of the preparation and stored at − 20 °C until use. Then, murine macrophages (RAW264.7; ATCC-TIB-71) (Manassas, VA, USA) were cultured Dulbecco’s modified Eagle medium (DMEM) (Thermo Fisher Scientific) supplemented with 10% heat-inactivated fetal bovine serum and 1% Penicillin–Streptomycin at 37 °C under 5% CO_2_ before use. After that, the macrophages at 1 × 10^5^ cells/well in 6-well plates were incubated for 24 h with several conditions (DMEM, inulin, Bifido, or Bifido with inulin) with or without LPS, using the LPS from Escherichia coli 026:B6 (Sigma-Aldrich, St. Louis, MO, USA) at 100 ng/ml. Notably, the dose of LPS to activate macrophages used here was followed our previous protocol^18–20^. After that, the expressions of several markers were measured by real time-qualitative polymerase chain reaction (RT-qPCR) using the primer presented in Table 1 with an established protocol. The RNA was extracted from the cells with TRIzol Reagent (Invitrogen, Carlsbad, CA, USA) together with RNeasy Mini Kit (Qiagen, Hilden, Germany) as 1 mg of total RNA was used for cDNA synthesis with iScriptreverse transcription supermix (Bio‐Rad, Hercules, CA, USA). Quantitative real‐time PCR was performed on a QuantStudio 5 real‐time PCR system (Thermo Fisher Scientific, Waltham, MA, USA) using SsoAdvanced Universal SYBR Green Supermix (Bio‐Rad, Hercules, CA, USA). Expression values were normalized to Beta‐actin (*β‐actin*) as an endogenous housekeeping gene and the fold change was calculated by the ∆∆Ct method. Additionally, supernatant cytokines of macrophage pro-inflammatory activity, including tumor necrosis factor–α (TNF-α), IL-6, and IL-1β, alone with anti-inflammatory function, including transforming growth factor-β (TGF-β) and IL-10, were also evaluated by ELISA assay followed the manufacturer’s protocols (R&D Systems, Inc., Minneapolis, MN, USA).

**Table 1:** Lists of primers used in the study.

Name	Forward	Reverse
Inducible nitric oxide synthase *(iNOS)*	5′-ACCCACATCTGGCAGAATGAG-3′	5′-AGCCATGACCTTTCGCATTAG-3′
Interleukin-1β *(IL-1β)*	5′-GAAATGCCACCTTTTGACAGTG-3′	5′-TGGATGCTCTCATCAGGACAG-3′
Tumor necrosis factor α *(TNF-α)*	5′-CCTCACACTCAGATCATCTTCTC-3′	5′-AGATCCATGCCGTTGGCCAG-3′
Arginase-1 *(Arg-1)*	5′-CTTGGCTTGCTTCGGAACTC-3′	5′-GGAGAAGGCGTTTGCTTAGTT-3′
Interleukin-10 *(IL-10)*	5′-GCTCTTACTGACTGGCATGAG-3′	5′-CGCAGCTCTAGGAGCATGTG-3′
Nuclear factor kappa B *(NF-κB)*	5′-CTTCCTCAGCCATGGTACCTCT-3′	5′-CAAGTCTTCATCAGCATCAAACTG-3′
Resistin-like molecule-α1 *(FIZZ-1)*	5′-GCCAGGTCCTGGAACCTTTC-3′	5′-GGAGCAGGGAGATGCAGATGA-3′
Transforming growth factor-β *(TGF-β)*	5′-CAGAGCTGCGCTTGCAGAG-3′	5′-GTCAGCAGCCGGTTACCAAG-3′
*β-actin*	5′-CGGTTCCGATGCCCTGAGGCTCTT-3′	5′-CGTCACACTTCATGATGGAATTGA-3′

### Macrophage cell energy status (the extracellular flux analysis)

The extracellular flux analysis using Seahorse XFp Analyzers (Agilent, Santa Clara, CA, USA) with oxygen consumption rate (OCR) and extracellular acidification rate (ECAR) representing mitochondrial function (respiration) and glycolysis activity, respectively, were determined in the 24 h-activated cells. Briefly, the stimulated macrophages at 1 × 10^5^ cells/well were incubated by Seahorse media (DMEM complemented with glucose, pyruvate, and l-glutamine) (Agilent, 103575-100) for 1 h before activation by different metabolic interference compounds, including oligomycin, carbonyl cyanide-4-(trifluoromethoxy)-phenylhydrazone (FCCP) and rotenone/antimycin A, for OCR evaluation. In parallel, glycolysis stress tests were performed using glucose, oligomycin, and 2-Deoxy-d-glucose (2-DG) for ECAR measurement. The data were analysed by Seahorse Wave 2.6 software based on the following equations: (i) maximal respiration = OCR between FCCP and rotenone/antimycin A - OCR after rotenone/antimycin A; (ii) maximal glycolysis (glycolysis capacity) = ECAR between oligomycin and 2-DG - ECAR after 2-DG.

### Statistical analysis

Wilcoxon signed-rank test was used to evaluate the difference in the change of variable outcomes between baseline and the 6th month, which included muscle biomarkers, IL-15 and creatinine cystatin C ratio, in clinical phase. The gene expressions of inflammatory and anti-inflammatory markers in macrophage cell lines, presenting with or without LPS, among the *Bifidobacterium* and inulin controls, and treated with *Bifidobacterium* + inulin were evaluated using One-way ANOVA with Tukey analysis for in vitro phase. The alpha level of 0.05 was considered statistically significant for all analyses. All analyses were conducted using SPSS version 28.0 (SPSS Inc., Armonk, NY, USA) and GraphPad Prism version 9.0 (GraphPad Software, Boston, MA, USA).

## Results

### The change in biomarkers of muscle building in clinical study

The stored plasma and serum samples from the children with obesity who received inulin supplementation (mean age: 10.3 ± 2.1 years, 54% male), placebo, and dietary fiber advice were analyzed. As mentioned above, in our previously published RCT of supplementation with inulin on body composition, we found that inulin supplementation increased fat-free mass in children with obesity^13^.

**Figure 1:**
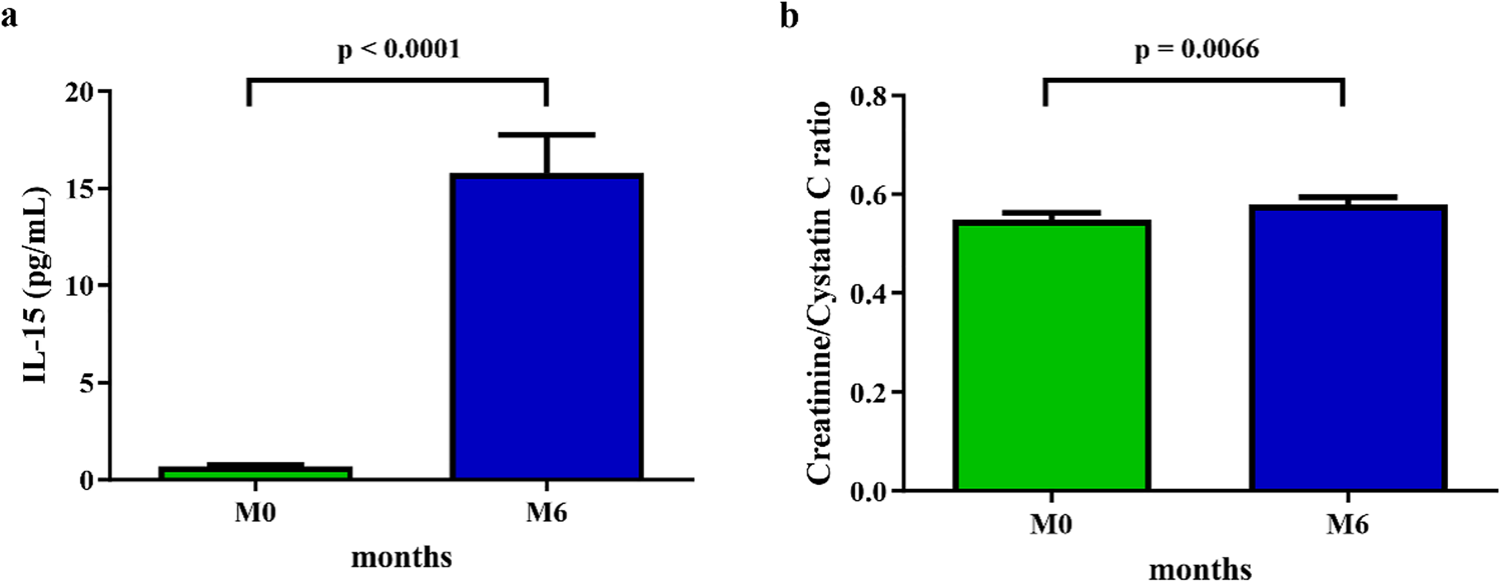
Change of IL-15 and serum creatinine/cystatin C ratio in the inulin group. Within group analysis showed significant increased IL-15 and creatinine/cystatin C ratio from the baseline only in the inulin group using Wilcoxon signed-rank test (p < 0.0001, 95% CI 10.9–18.6, and p = 0.0066, 95% CI 0.005–0.05, respectively) (n = 46). *IL* interleukin.

IL-15, a myokine linked to muscle building, significantly increased in the inulin group (p < 0.0001, 95% CI 10.9–18.6). Moreover, within group analysis showed that (p = 0.0066, 95% CI 0.005–0.05) (Fig. 1a,b). The changes of IL-15 and creatinine/cytatin C ratio in the placebo and dietary fiber advice groups were not observed.

### The change in pro-inflammatory and anti-inflammatory macrophage responses in cell line study

**Figure 2:**
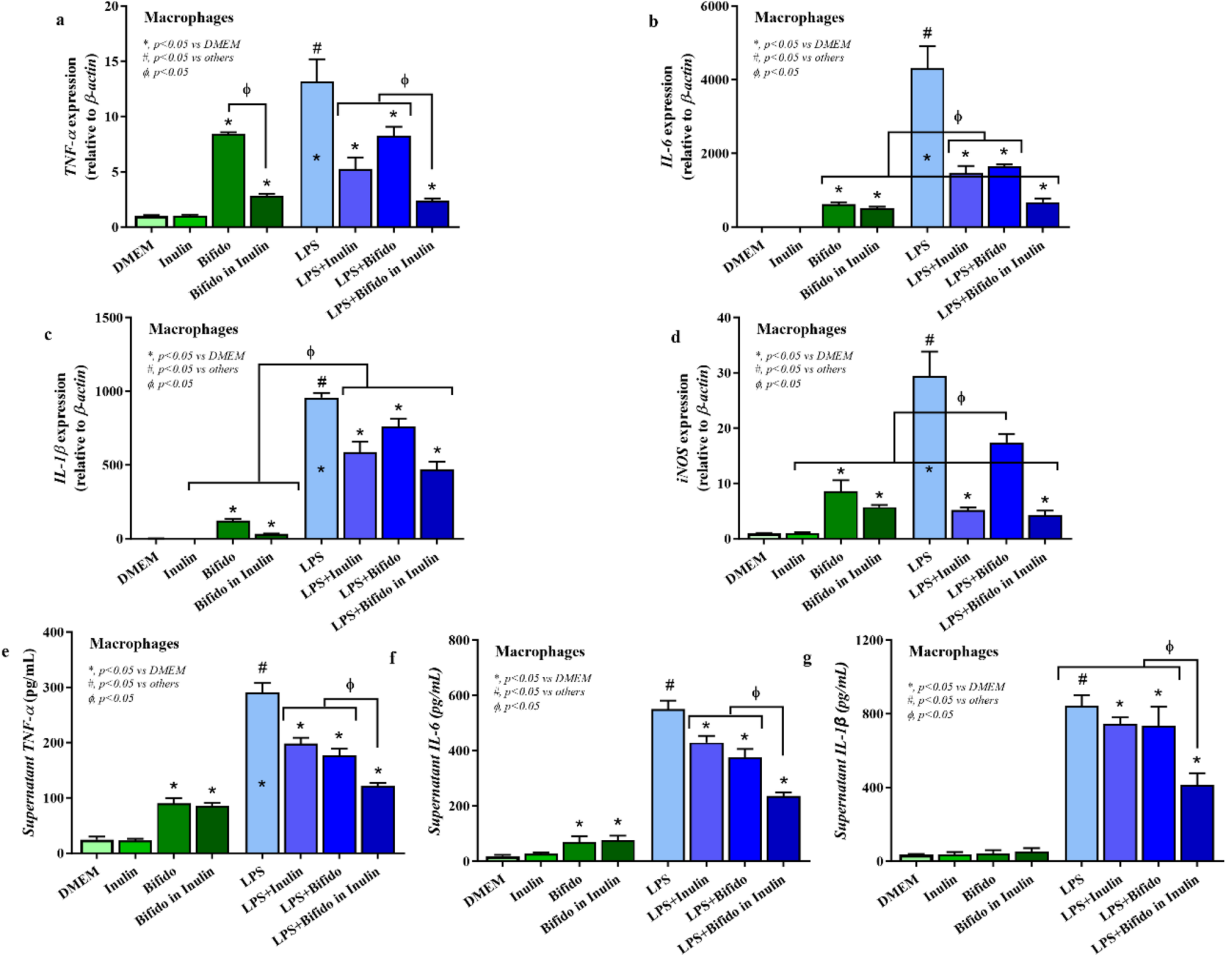
Pro-inflammatory macrophage responses. In the presence of LPS, treatment of the cell-free supernatant from *Bifidobacterium* + inulin significantly decreased expression of *TNF-α*, *IL-6*, *IL-1β*, and *iNOS* compared to the inulin and *Bifidobacterium* controls, and the LPS (**a**–**d**) (p < 0.05). Supernatant pro-inflammatory cytokines (TNF-α, IL-6, and IL-1β) was also significantly lower than LPS control (**e**–**g**) using One-way ANOVA with Tukey analysis (p < 0.05). *IL* interleukin, *iNOS* inducible nitric oxide synthase, *LPS* lipopolysaccharide, *TNF* tumor necrosis factor.

**Figure 3:**
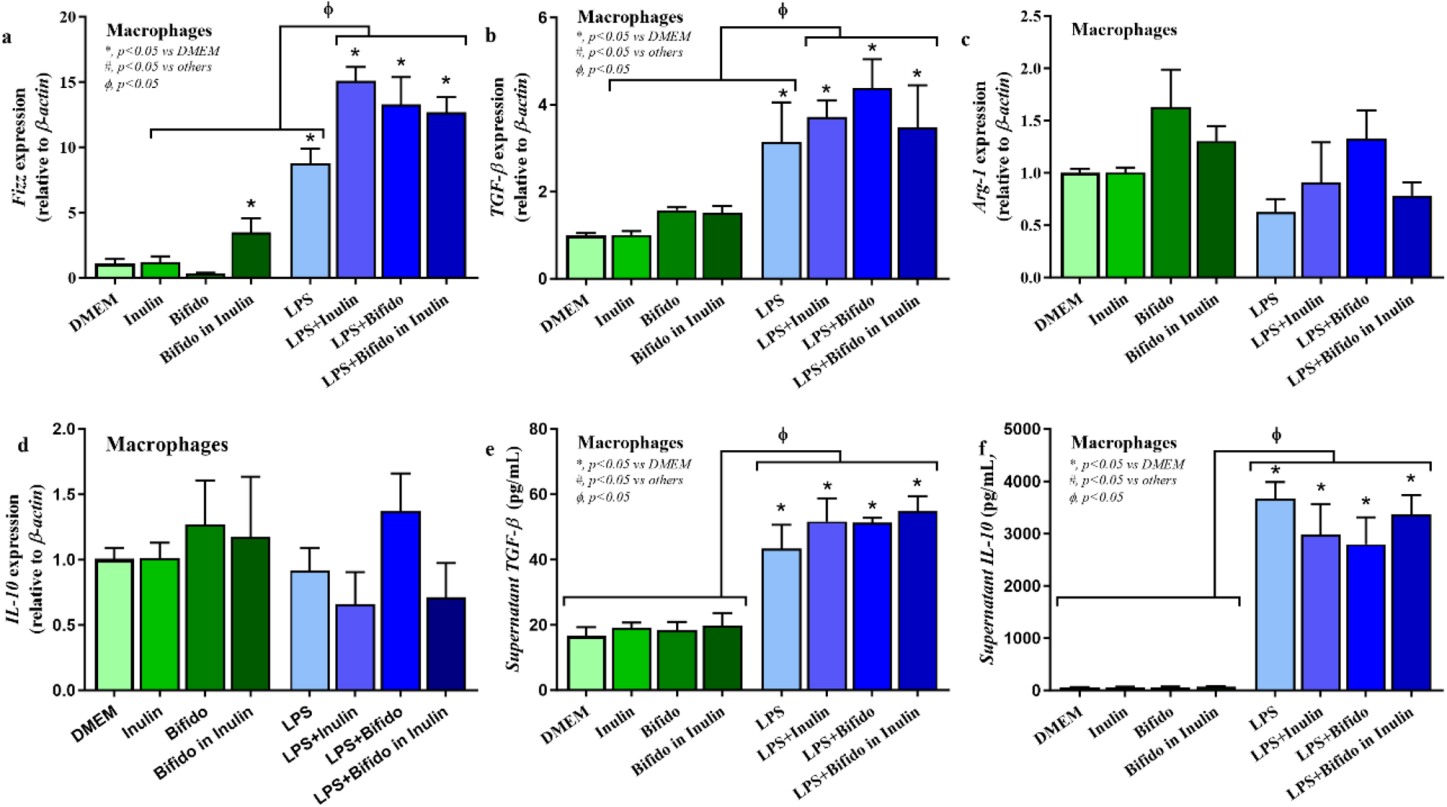
Anti-inflammatory macrophage responses. Treatment from *Bifidobacterium* + inulin significantly upregulated gene expression of *FIZZ-1* and *TGF-β*, but not *Arg-1* and *IL-10* compared to the LPS (**a**–**d**). No alteration of supernatant anti-inflammatory cytokines (TGF-β and IL-10) was observed after *Bifidobacterium* + inulin administration compared with LPS control (**e**,**f**) using One-way ANOVA with Tukey analysis (p < 0.05). *Arg*, arginase, *IL* interleukin, *TGF* transforming growth factor.

**Figure 4:**
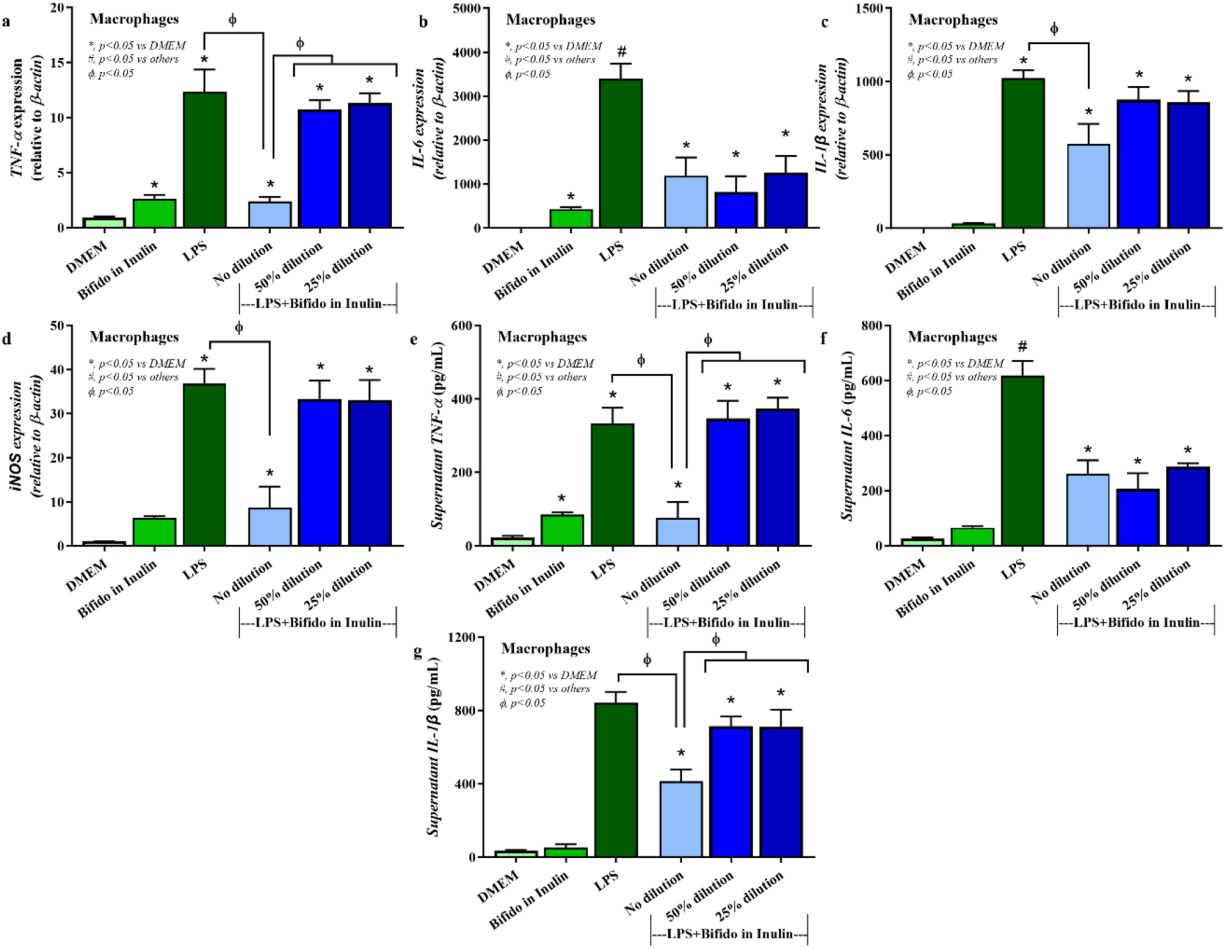
Dose effect of *Bifidobacterium longum* fermentative inulin (Bifido in Inulin or *Bifidobacterium* + inulin) for macrophage responses using no dilution, 50% dilution, and 25% dilution by culture media (DMEM). Compared to LPS group, treatment with *Bifidobacterium* + inulin without dilution significantly downregulated gene expression of *TNF-α*, *IL-6, IL-1β, and iNOS* and also reduced production of supernatant cytokines (*TNF-α*, *IL-6,* and *IL-1β*), while the dilution attenuated only IL-6 expression and supernatant IL-6 (**a**–**g**) as calculated by One-way ANOVA with Tukey analysis (p < 0.05). *IL* interleukin, *iNOS* inducible nitric oxide synthase, *LPS* lipopolysaccharide, *TNF* tumor necrosis factor.

Our in vitro phase demonstrated that metabolites derived from *Bifidobacterium* capable of utilizing inulin contained the abundance of short chain fatty acids (SCFAs) (Supplement Fig. 1). In the presence of LPS, treatment of the cell-free supernatant from *Bifidobacterium* + inulin significantly downregulated pro-inflammatory genes, including TNF-α and IL-6 expression compared to the media control, *Bifidobacterium* alone, inulin alone, and the LPS (p < 0.05) (Fig. 2a,b). Downregulation of *IL-1β* and inducible nitric oxide synthase (*iNOS*) after *Bifidobacterium* + inulin administration was also significantly different from the LPS (p < 0.05) (Fig. 2c,d). Although inulin and *Bifidobacterium* supernatant alone reduced supernatant TNF-α and IL-6 compared to LPS control, *Bifidobacterium* + inulin further decreased both cytokines (p < 0.05) (Fig. 2e,f). On the other hand, only *Bifidobacterium* + inulin but not each factor in separation attenuated LPS-activated IL-1β in macrophages (p < 0.05) (Fig. 2g). For the anti-inflammatory responses, treatment from *Bifidobacterium* + inulin significantly upregulated *FIZZ-1* and *TGF-β* expression compared to the LPS (p < 0.05) (Fig. 3a,b). No significant upregulation of,*Arginase-1*, and *IL-10* expression (Fig. 3c,d) and alteration of supernatant anti-inflammatory cytokines (TGF-β and IL-10) (Fig. 3e,f) was observed after *Bifidobacterium* + inulin administration compared with LPS control. Dose effect of *Bifidobacterium longum* fermentative inulin (Bifido in Inulin or *Bifidobacterium* + inulin) for macrophage responses was performed using no dilution, 50% dilution, and 25% dilution by culture media (DMEM). Compared to LPS group, treatment with *Bifidobacterium* + inulin without dilution significantly downregulated gene expression of *TNF-α*, *IL-6, IL-1β, and iNOS* and reduced production of supernatant cytokines (*TNF-α*, *IL-6,* and *IL-1β*), while the dilution attenuated only *IL-6* expression and supernatant IL-6 (Fig. 4a–g). The signal pathway diagram of the relationship between inflammation and microbiota-muscle axis was illustrated in Supplement Fig. 2.

### The change in cell energy status in cell line study

**Figure 5:**
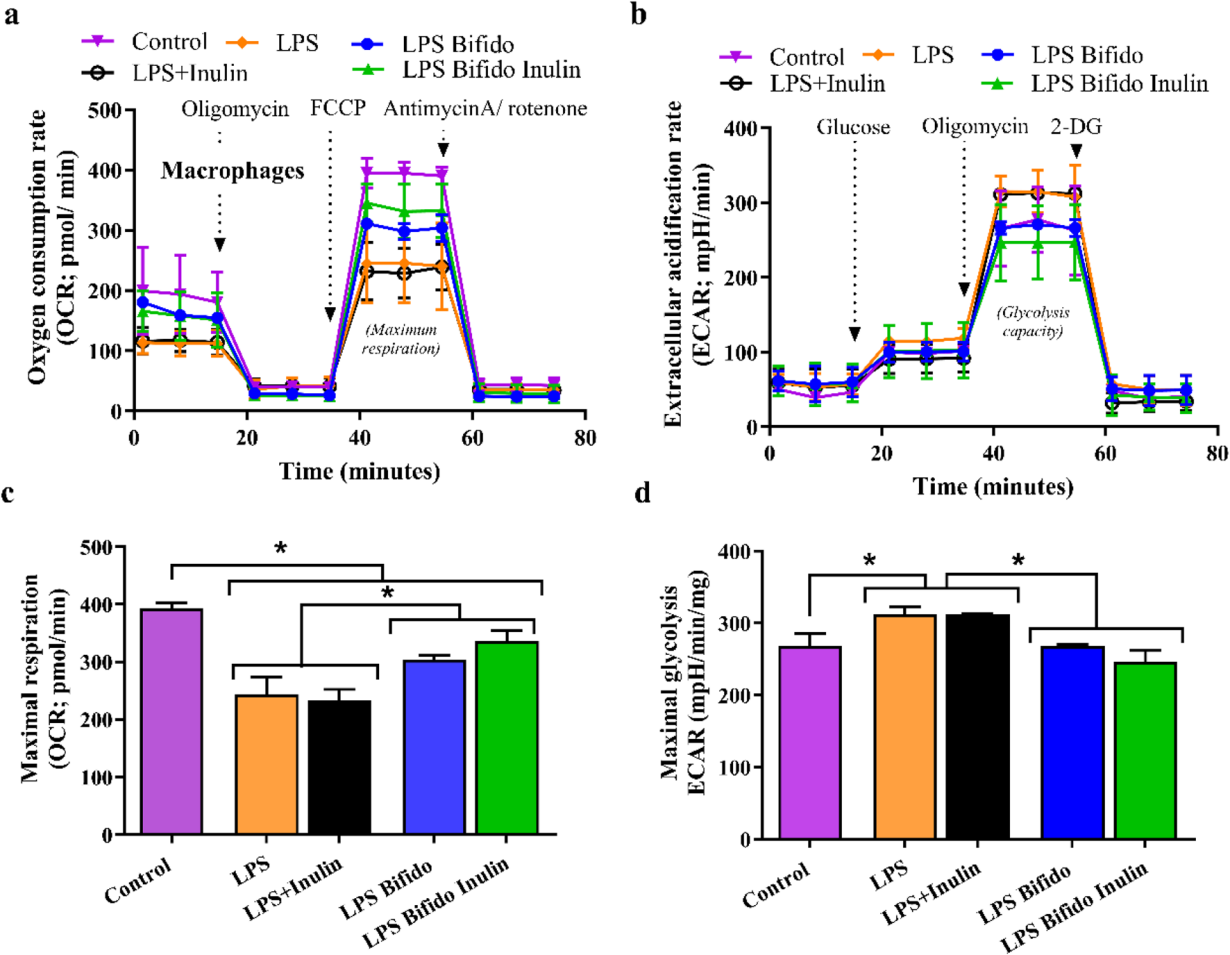
Cell energy status. All activations reduced mitochondrial activities as indicated by maximal respiration (most prominence in LPS and LPS plus inulin), while only LPS and LPS plus inulin elevated glycolysis (p < 0.05). One-way ANOVA with Tukey analysis was used to evaluate parametric variables. Macrophages, RAW264.7 murine cell line; Oligomycin, an inhibitor against mitochondrial ATP synthesis; Antimycin A, a respiratory complex III inhibitor; Rotenone, a respiratory complex I inhibitor; 2-DG (2-Deoxy- d-glucose), a glycolysis inhibitor.

Because of a possible relationship between cell energy status and macrophage functions, mitochondrial and glycolysis activities were determined through oxygen consumption rate (OCR) and extracellular acidification rate (ECAR), respectively (Fig. 5a,b). For mitochondrial activities, the OCR of macrophages was decreased by all activations; however, LPS and LPS plus inulin demonstrated the most severe reduction of mitochondrial functions (maximal respiration) (Fig. 5c). The mitochondrial activities were improved with the presence of *Bifidobacterium* supernatant regardless of inulin (Fig. 5c), implying some beneficial molecules from the probiotics. For glycolysis status, only LPS and LPS plus inulin elevated glycolysis (Fig. 5d) which might be correlated with LPS-induced pro-inflammatory macrophage activity (Fig. 2). Nevertheless, the LPS-enhanced maximal glycolysis was reduced into the control regular state with incubation by supernatant of *Bifidobacterium*, alone or with inulin (Fig. 5d). In comparison with the control, the LPS-induced glycolytic stress effect in *Bifidobacterium* and *Bifidobacterium* + inulin supernatants was not observed (Fig. 5d), suggesting the influence of some bifidobacterial molecules on glycolysis.

## Discussion

Obesity is a worldwide health problem and links with dysbiosis of the gut microbiota^1,7^. Pediatric obesity is associated with compromised muscular capacity and fitness^21^. Sarcopenic obesity is an arising issue in this population characterized by a significant decline in muscle mass alongside excessive fat accumulation^22^. We studied the benefits of the inulin extracted from Thai Jerusalem artichoke on children with obesity and found a significant increase in fat-free mass after a 6-month period of inulin supplementation^13^. In the present study, we examined the mechanism underlying gut-muscle axis from inulin supplementation on muscle mass in children with obesity. The in vivo study found a significant increase in IL-15 and creatinine/cystatin C ratio in the inulin supplementation group. For the in vitro study of inflammatory and anti-inflammatory gene expression, a significant downregulation of TNF, IL-6, IL-1β, and iNOS expression in the treatment with *Bifidobacterium* plus inulin was observed, whereas a significant upregulation of FIZZ-1 and TGF-β as the anti-inflammatory gene expressions was found. Furthermore, there were no significant differences in cellular glycolytic capacity between the treatment with *Bifidobacterium* plus inulin and controls.

Several studies in adults showed that supplementation with prebiotics, probiotics, and synbiotics improved muscle mass and strength^23–27^. Some studies were conducted on animal models to examine muscle mass and strength, but they did not specifically explore muscular biomarkers^28,29^. As far as we are aware, there has not been research done in children regarding the effect of prebiotics on muscular biomarkers via gut-muscle axis; therefore, our study might be the first to support this hypothesis. IL-15 was proposed as a contraction-induced myokine acting on local skeletal muscle to improve energy metabolism^30^. O'Leary et al. found that IL-15 played a role in myogenesis and protected muscles from degradation in isolated human myogenic cultures^31^. Another study revealed that exercise leads to higher serum IL-15, contributing to improved physical fitness^32^. In addition, IL-15 has been investigated for its role in indicating cancer-induced cachexia and its potential to prevent sepsis-induced muscle wasting^33,34^. Serum creatinine/cystatin C ratio was also offered as a biomarker of skeletal muscle status^35^. Basically, creatinine and cystatin C are used for estimating glomerular filtration. In contrast to creatinine, which is solely a product of muscle catabolism, cystatin C is produced from all nucleated cells and remains unaffected by muscle mass. Kashani et al. firstly investigated the benefits of serum creatinine to cystatin C ratio, so called the sarcopenia index, for assessing muscle mass^36^. Studies have highlighted the potential of this ratio in predicting severity, muscle mass, muscle strength, and muscle-adjusted visceral fat mass in non-alcoholic fatty liver disease patients^37,38^. In the case of the elderly, the researchers found the usefulness of creatinine/cystatin C ratio in assessing sarcopenia in terms of muscle mass and strength^39^. Therefore, based on the previous literatures, all the proxies of myogenesis from our study indicated inulin-induced muscle mass production and corresponded with increased fat-free mass in the children with obesity.

Systemic inflammation contributes to organ damage, including skeletal muscle loss, thereby increasing the risk of sarcopenia in children with obesity^22^. The study by Kalinkovich et al. revealed that a muscle mass reduction was caused by adipose tissue inflammation along with decreased uptake of fatty acids which led to an increased deposition of lipids within skeletal muscle tissue. This, in turn, could result in a decline in skeletal muscle mass^40^. Another previous study illustrated that change in the composition of gut microbiota, resulting in heightened absorption of bacterial products like LPS, could induce chronic inflammation and muscle production through elevated levels of inflammatory cytokines such as IL-6 and TNF-α, and then TNF-α triggers the activation of NF-κB and muscle ring finger-1, which in turn promotes increased ubiquitination of muscle proteins. This cascade subsequently leads to the breakdown of actin and myosin myofilaments, culminating in a reduction of muscle mass^41^. Additionally, there are other potential pathways, such as the production of mediators by the gut microbiota, including SCFAs, which play a role in positively influencing skeletal muscle production. The administration of butyrate, recognized for its anti-inflammatory and muscle-building properties attributed to its ability to inhibit the enzyme histone deacetylase, facilitates improved muscle building^42^. Our in vitro study showed that metabolites produced by *Bifidobacterium*, which can utilize inulin, were rich in SCFAs, and for in vitro study in macrophage cell lines, treatment with *Bifidobacterium* together with inulin significantly downregulated the key inflammatory gene expression, TNF-α and IL-6, and significantly decreased the expression of M1 macrophage markers, IL-1β and iNOS. These findings align with a study by Nicolucci et al. which reported a significant reduction in IL-6 with inulin supplementation compared to the control group. However, contrary to our results, they found no significant difference in lean mass gain in the inulin group^10^. Kelishadi et al. found a significant reduction of TNF-α and IL-6 in the synbiotic supplementation group^43^. The finding corresponded to our study reflecting the potential of rebalancing the gut microbiota dysbiosis leading to decreased inflammation. Interestingly, *Bifidobacterium* in DMEM (the glucose containing culture media) demonstrated a less potent anti-inflammation than *Bifidobacterium* with inulin (Fig. 2e–g) suggesting an impact of the different metabolites secreted from the probiotics after an incubation by glucose and inulin. Indeed, inulin stimulates the growth and activity of lactic acid bacteria as a bacterial nutrient source resulting in production of SCFAs, a beneficial factor for enterocyte to strengthen gut permeability^44^. More mechanistic studies are interesting.

Although we found *Bifidobacterium* could be an important link to promote muscle mass gain via gut-muscle axis, this is not limited only to this bacterium. Previous study reported that *Bacteroides* was positively associated with parameter of muscle function^41^, while a decrease in the genus *Akkermansia* was found in the gut microbiota of sarcopenia patients with cirrhosis^45^.

Dysbiosis of the intestinal microbiota impairs the gut barrier, enabling toxic substances derived from pathogenic bacteria, such as LPS, to enter the bloodstream. Activation of TLR4 by LPS can increase the levels of NF-κB and proinflammatory cytokines, including IL-6 and TNF-α^46,47^. Additionally, an initiation of inflammatory cascade in obesity via M1 macrophage that contributes to increased M1 macrophage markers, IL-1β and iNOS, leading to increased systemic inflammation and then affects muscle mass production^15,48^. Without LPS, *Bifidobacterium* condition media (with or without inulin) mildly upregulated expression of IL-1β and iNOS indicated some degree of inflammatory responses, perhaps due to the pathogen-associated molecular patterns (PAMPs) of bacteria. On the other hand, with LPS, the *Bifidobacterium* condition media downregulated these genes supporting the possible secretion of anti-inflammatory molecules. In the future, the separation of the anti-inflammatory fraction from *Bifidobacterium* culture media might be interesting. However, inulin did not show inflammatory response because *Bifidobacterium* combined with inulin without LPS was not different from the *Bifidobacterium* culture media alone. Moreover, inulin itself might have an anti-inflammatory response because inulin alone with LPS downregulated IL-1β and iNOS (the genes of M1-pro-inflammatory polarization)^49^ and upregulated FIZZ-1 and TGF-β, the anti-inflammatory M2 macrophage polarization genes^50^. Perhaps, inulin ameliorates LPS-TLR4 on macrophages by inhibiting their adhesion, leading to reduced inflammation^49^ and promotes M2 macrophage polarization. In addition, SCFAs, induced by inulin might attenuate inflammation through increased mitochondrial function and muscle building^51^ as indicated by increased fat-free mass and muscle biomarkers in our previous RCT^13^ and this present study. Moreover, LPS reduced mitochondrial function and elevated glycolysis (extracellular flux analysis) supported the association between energy status and macrophage polarization^52^ which was normalized by *Bifidobacterium* condition media. Because the cell energy status using *Bifidobacterium* with or without inulin was not different, inulin did not have an additional impact on cell energy status that was already improved by *Bifidobacterium*. Probiotic-induced anti-inflammatory macrophages might partly attenuate obesity-induced systemic inflammation through the strengthening of gut permeability^18–20^.

Indeed, there are several evidences of probiotic-induced anti-inflammation. For instance, *Lactobacillus acidophilus* was shown to counter inflammation through TGF-β1 signalling after Salmonella infection^53^. Fujii et al. investigated the anti-inflammatory properties of *Bifidobacterium breve* supplementation in preterm infants, which resulted in an observable augmentation in serum TGF-β1 levels^54^. Taken together, these might support our results of the anti-inflammatory role of prebiotics and probiotics, as we observed a trend towards elevated the gene expression of anti-inflammatory biomarkers, FIZZ-1 and TGF-β, in the current study. Furthermore, prebiotics like inulin might stimulate anti-inflammatory responses through alternative pathways. The incorporation of additional anti-inflammatory markers, such as peroxisome proliferator-activated receptor gamma, could yield supplementary evidence and further strengthen the findings^55^.

To the best of our knowledge, this is the first study regarding the gut-muscle axis in children with obesity. The research was meticulously designed to elucidate the mechanisms underlying prebiotic-induced muscle building. All the results in myogenesis were the novel findings which were not found in the previous studies of prebiotics in obese children. Another strength is the method used in the study. We performed a clinical study according to our previous RCT study and the study was done in human subjects enhancing the potential for future practical applications.

In conclusion, the supplementation of inulin significantly promoted the biomarkers of muscle building in agreement with fat-free mass gain in children with obesity. This could be explained by *Bifidobacterium* metabolites derived from inulin digestion exhibited in vitro anti-inflammatory activity. Those metabolites may decrease systemic pro-inflammation, thus promoting muscle production in children with obesity via gut-muscle axis response. To bolster these results, future studies incorporating inulin alongside multi-strain probiotics may uncover further insights into the interaction between gut microbiota and muscle health in children with obesity.

### Supplementary Information

Supplementary Information.

## Data Availability

Data described in the manuscript will be made available upon request pending application and approval from the corresponding author.
