# Supplementary material for: Inulin supplementation exhibits increased muscle mass via gut-muscle axis in children with obesity: double evidence from clinical and in vitro studies
Source: Sci Rep. 2024 May 16;14:11181. doi: 10.1038/s41598-024-61781-1 (PMC11099025; doi:10.1038/s41598-024-61781-1)
Supplement: Supplementary file 1 — Supplementary Information. [file 41598_2024_61781_MOESM1_ESM.pdf]

SCFA analysis
:Acetic acid
:Propionic acid
:Isobutyric acid

Bacteria
:BL02 = B. longum
:LJ1386 = L. johnsonii
:ED1543 = E. durans
:EF2165 = E. faecium

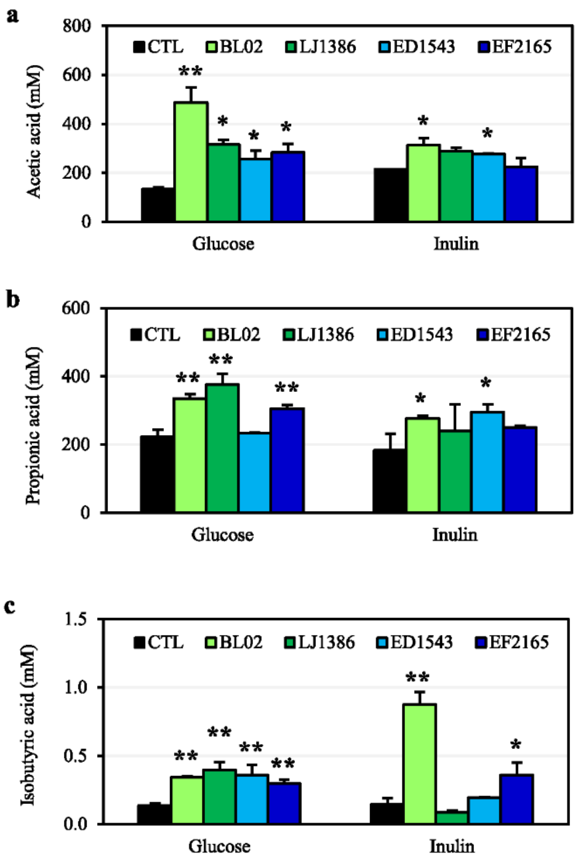

Supplement Figure 1 Metabolites derived from *Bifidobacterium longum* capable of utilizing inulin contained the abundance of SCFAs, including (a) acetic acid, (b) propionic acid, and (c) isobutyric acid. *Bifidobacterium longum* with inulin significantly enhanced acetic acid, propionic acid, and isobutyric acid compared to the control using One-way ANOVA with Tukey analysis (*p < 0.05 and **p < 0.01).

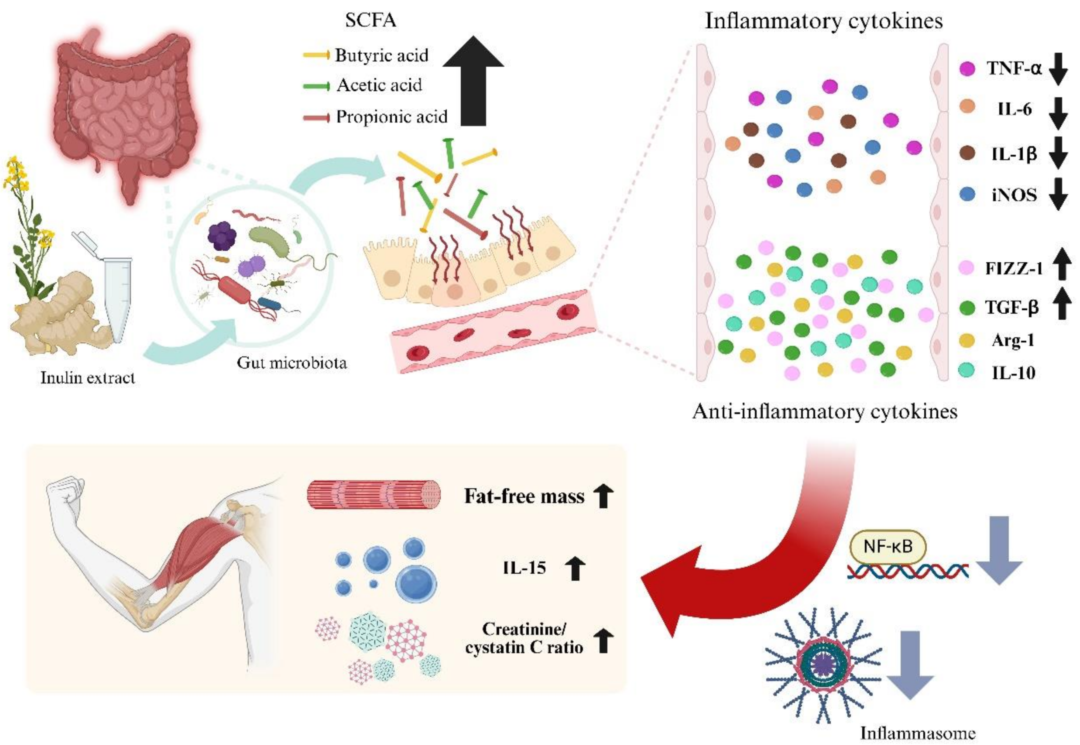

Supplement Figure 2 The signal pathway diagram of the relationship between inflammation and microbiota-muscle axis. Inulin supplementation enhanced the muscle building biomarkers, IL-15 and creatinine/cystatin C ratio, and promoted fat-free mass, elucidating by *Bifidobacterium* metabolites derived from inulin digestion which demonstrated anti-inflammatory activity by increasing FIZZ-1 and TGF-β, while decreasing systemic pro-inflammation, TNF-α, IL-6, IL-1β, and iNOS. This cascade led to the attenuation of NF-κB activation and the inflammasome pathway, thus promoting muscle production through gut-muscle axis response.

Arg, arginase; IL, interleukin; iNOS, inducible nitric oxide synthase; LPS; lipopolysaccharide; NF-κB, nuclear factor-κB; SCFA, short chain fatty acid; TGF, transforming growth factor; TNF, tumor necrosis factor
